# Impact of school policies on non-communicable disease risk factors – a systematic review

**DOI:** 10.1186/s12889-017-4201-3

**Published:** 2017-04-04

**Authors:** Ankur Singh, Shalini Bassi, Gaurang P. Nazar, Kiran Saluja, MinHae Park, Sanjay Kinra, Monika Arora

**Affiliations:** 1 Australian Research Centre for Population Oral Health (ARCPOH), Adelaide Dental School, The University of Adelaide, Adelaide, Australia; 2 Health Promotion Division, Public Health Foundation of India (PHFI), Plot No. 47, Sector 44, Gurgaon, Haryana 122002 India; 3 Department of Non-Communicable Disease Epidemiology, Faculty of Epidemiology and Population Health, London School of Hygiene and Tropical Medicine, London, UK

**Keywords:** Non-communicable disease, School policy, Systematic review, NCD risk factor

## Abstract

**Background:**

Globally, non-communicable diseases (NCDs) are identified as one of the leading causes of mortality. NCDs have several modifiable risk factors including unhealthy diet, physical inactivity, tobacco use and alcohol abuse. Schools provide ideal settings for health promotion, but the effectiveness of school policies in the reduction of risk factors for NCD is not clear. This study reviewed the literature on the impact of school policies on major NCD risk factors.

**Methods:**

A systematic review was conducted to identify, collate and synthesize evidence on the effectiveness of school policies on reduction of NCD risk factors. A search strategy was developed to identify the relevant studies on effectiveness of NCD policies in schools for children between the age of 6 to 18 years in Ovid Medline, EMBASE, and Web of Science. Data extraction was conducted using pre-piloted forms. Studies included in the review were assessed for methodological quality using the Effective Public Health Practice Project (EPHPP) quality assessment tool. A narrative synthesis according to the types of outcomes was conducted to present the evidence on the effectiveness of school policies.

**Results:**

Overall, 27 out of 2633 identified studies were included in the review. School policies were comparatively more effective in reducing unhealthy diet, tobacco use, physical inactivity and inflammatory biomarkers as opposed to anthropometric measures, overweight/obesity, and alcohol use. In total, for 103 outcomes independently evaluated within these studies, 48 outcomes (46%) had significant desirable changes when exposed to the school policies. Based on the quality assessment, 18 studies were categorized as weak, six as moderate and three as having strong methodological quality.

**Conclusion:**

Mixed findings were observed concerning effectiveness of school policies in reducing NCD risk factors. The findings demonstrate that schools can be a good setting for initiating positive changes in reducing NCD risk factors, but more research is required with long-term follow up to study the sustainability of such changes.

**Electronic supplementary material:**

The online version of this article (doi:10.1186/s12889-017-4201-3) contains supplementary material, which is available to authorized users.

## Background

Non-communicable diseases (NCDs) cause about 40 million deaths each year globally [1]. The four most important modifiable behavioral risk factors for NCDs include unhealthy diet, physical inactivity, tobacco use [2, 3] and harmful use of alcohol [4]. According to the estimates from the most recent Global Burden of Disease study, out of all the deaths due to NCDs in 2015, approximately 12 million deaths were due to unhealthy diet, 6.5 million were due to tobacco use, 1.8 million were due to alcohol and drug use and 1.6 million deaths were attributed to low physical activity [1]. The major risk factors for NCDs are associated with behavioral patterns that are largely established during childhood and adolescence and continue into adulthood [5–7]. The onset of many NCDs like diabetes, obesity, and cardiovascular diseases can be prevented or delayed by addressing these risk factors earlier in life [8].

Children and adolescents should be prioritized as target groups for behavioral interventions due to their high adaptability and likelihood to be motivated for appropriate healthy modifications [9]. In support of this, evidence shows that behavioral modifications are more successful if implemented at an early stage [10, 11]. Behavioral changes during early years require conducive policies and programs [12]. Hence, in addition to prioritizing children for the adoption of healthy behavioral practices, they should be provided with a supportive environment for behavior change in settings where children live, play and study [13].

Schools are uniquely positioned as ideal settings to model, promote and reinforce healthy behaviors among children and adolescents. Children and adolescents spend much of the daytime at school and can easily access the schools’ health-related educational programs. Therefore, schools function as health hubs by educating and imparting healthy habits among students [14, 15] as they service a large population of children and adolescents [7]*.* Evidence suggests that school policies can positively impact Body Mass Index (BMI) [16], physical activity and dietary behaviors [17] among children.

Previous studies have mostly looked at the relationship between school policies and specific risk factors. There exists no review that has systematically identified and collated evidence on the effectiveness of school-based policy interventions collectively for the four major preventable NCD risk factors (unhealthy diet, physical inactivity, tobacco use and alcohol use). Furthermore, no systematic review has examined the impact of school policies on the anthropometric & physiological measures in children. Therefore, the aim of this systematic review was to identify, collate and synthesize the existing literature on the impact of school policies on major risk factors of NCDs.

## Methods

### Search strategy and study selection criteria

A review protocol was developed in accordance with PRISMA guidelines [18]. The search strategy aimed to identify published articles on the effectiveness of school level policy interventions to reduce major preventable risk factors for NCDs (unhealthy diet, physical inactivity, tobacco use, alcohol use, excess body weight, high blood pressure, adverse lipid profile as well as anthropometric and physiological measures) among students. The search was carried out in three electronic databases: Ovid Medline, EMBASE, and Web of Science. The search strategy used to identify the studies in Medline is included in Additional file 1.

The databases were searched for studies published from January 1990 to January 2014. The inclusion criteria were established to include studies assessing effectiveness of either existing or new school based policy interventions among children between the age of 6 to 18 years aimed at the reduction of NCD risk factors. Studies that assessed the effectiveness of pre-school policy intervention were excluded. The detailed inclusion and exclusion criteria guiding the selection of studies for the review is described in Table 1. Duplicate references were removed using software (Endnote X7), and titles and abstracts were independently screened by two reviewers (AS and SB). Any disagreements were resolved by discussion and consultation with a third investigator (MA). Following this step, full text of the selected studies were retrieved and then reviewed for relevance to the inclusion and the exclusion criteria by AS and SB independently. Disagreements at this stage were resolved through discussion between the two reviewers.

**Table 1:** Inclusion and exclusion criteria

Inclusion Criteria	Exclusion Criteria
Population Children or adolescents between the age-group of 6 – 18 years.	Population Children or adolescents not in the specified age-group and studies conducted on animal models.
Intervention Policies that modify the four identified risk factors (unhealthy diet, physical inactivity, alcohol and tobacco use) and associated health related behaviors amongst the students either alone or as part of any intervention program.	Intervention Policy components those are insufficiently described to enable replication. School policies focusing on differently abled students.
Context Schools as a setting.	Context Community, pre-schools and clinical settings.
Outcome Prevalence of health related behaviors identified as risk factors for NCDs.	Study design Editorials, library thesis, opinions and letters, papers with insufficient methodological details reported to allow critical appraisal of study quality, studies not in English language. Studies published before 1990.
Study Design Any experimental or observational study design (randomized controlled trial controlled before-after study, quasi-experimental, interrupted time series, cohort study or cross-sectional study).	

### Data extraction

Two reviewers (AS and SB) independently extracted information from the selected papers using pre-piloted data extraction forms. Any disagreements were resolved either by discussion or by the intervention of another investigator (MA). The following data were extracted: study characteristics (primary author, year of publication, study setting, age group, sub-groups analysed, sample size, data collection methods, inclusion criteria, randomization information, statistical analysis); intervention or policy component, study outcomes (primary outcomes: BMI, waist circumference, overweight, obesity, physical activity, tobacco use, alcohol use, other relevant outcomes; secondary outcomes: knowledge and attitude), type of effect estimates, main result and statistical significance of differences.

### Quality assessment

All the papers included in the review were independently assessed for methodological quality using the Effective Public Health Practice Project (EPHPP) quality assessment tool [19] by AS and then cross-checked by SB. The EPHPP tool contains eight different components but the scoring on quality assessment is done by six parameters. These include selection bias, study design, identification and treatment of confounders, blinding, data collection methods and withdrawals and dropouts. The components were rated strong, moderate, or weak according to a standardized guide and corresponding guidelines in the dictionary. Those with no weak ratings and at least four strong ratings were considered ‘Strong.’ Those with less than four strong ratings and one weak rating were considered ‘Moderate.’ Finally, those with two or more weak ratings were considered ‘Weak.’ The two remaining components within the quality assessment included in the assessment were the integrity of the intervention and the use of appropriate analysis [19].

### Synthesis of evidence

Due to the heterogeneity in policy components of the interventions included, outcomes and effect measures, a meta-analysis was not considered appropriate. A description of effectiveness measures and a narrative review were considered appropriate to present the findings of the study.

## Results

**Fig. 1:**
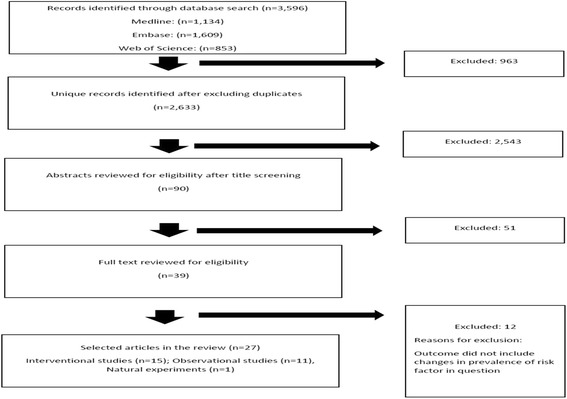
Flowchart for study identification and selection process

Overall, 27 studies were included in the review after the full-text screening of the identified articles through systematic database searching (*n* = 2633), title and abstract screening (*n* = 90), application of inclusion and exclusion criteria (*n* = 39) and full-text review (Fig. 1).

The majority of included studies were from high-income countries, USA (15), Australia (4), UK (2), Canada (2), Spain (1), Greece (1), combined USA and Australia (1) with the exception of only one study from India (1). There were 15 interventional studies (eight randomized controlled trials (RCTs), seven quasi-experimental studies), 11 observational studies (ten cross-sectional studies, one case-control) and one natural experiment. Five out of 27 studies were based in schools from socioeconomically deprived areas. Of the 27 studies, ten assessed the effectiveness of multiple policy interventions and 14 studies evaluated multiple outcomes (physical measures, biomarker levels and behaviors). The remaining three studies only assessed single intervention or outcome. Collectively, the children within the selected studies ranged from 6 to 17 years and were in grades from 1st to 12th. Apart from one study [20] which included only boys, remaining studies included both boys and girls. Based on the quality assessment of the selected studies, 18 were categorized as having weak methodological quality, six with moderate quality and three with strong methodological quality (Table 2; Additional file 2).

**Table 2:** Descriptive summary of the included studies

S/No	Study	Year	Country	Study Design	Sample size (n)	Participant Inclusion Criteria	Policy/ Policy Intervention	Outcome/s measured	Quality
1	Anthamatten et al.	2011	USA	Observational study; case-control	*n* = 3688	Participation in Learning Landscape Program; recent schoolyard renovation, the size of the school, and the social and demographic characteristics of the school population.	Physical Activity (learning landscape program)(Renovation of school grounds)	Utilization of school yards for Physical Activity	Weak
2	Blum et al.	2008	USA	Quasi-experimental	*n* = 456 students from 7 schools;	Students from grade 9-11	Elimination from SSB (Diet) and other junk food in schools food policy	Change in students beverage servings/day	Weak
3	Covelli et al.	2008	USA	Quasi-experimental - Repeated Measures	*n* = 48 (Intervention = 31; control = 17)	(1) be of African-American ethnicity, (2) be between 14 and 17 years old, (3) be able to read and write in English, (4) have obtained a signed parental/guardian consent form, and (5) have signed a participant assent form.	Integration of health promotion in existing curriculum (Provision of cognitive behavioral components of health knowledge, health promotion concepts, nutrition, and exercise).	Health promotion knowledge, behaviors related to fruit and vegetable intake and exercise; blood pressure	Weak
4	Evans-Whipp et al.	2010	USA	Cross-sectional	*n* = 3466 from 285 schools	One class per school was invited to take part in the study. Selected classes were from three-year levels: Grade 5 (age 10), Year 7 (age 12) and Year 9 (age 14).	Existing School tobacco policies (Washington and Victoria) - Comprehensive smoking bans, policy orientation towards abstinence and harm minimization principles, possession of tobacco products among students	Current tobacco use; daily tobacco use; students perception about school smoking	Moderate
5	Evans-Whipp et al.	2013	USA and Australia	Cross-sectional	*n* = 1848	Students from grade 5,7 or 9	School alcohol policies (IYDS)	Current alcohol use; alcohol use in schools ground	Moderate
6	Foster et al.	2008	USA	RCT	*n* = 10 schools; *n* = 1349 students	Not mentioned	School self-assessment; nutrition education; nutrition policy; social marketing; and parent outreach.	Sales of lower-fat à la carte foods; lower fat food choices; fruit and vegetable intake; Environmental and behavioral perceptions	Strong
7	French et al.	2004	USA	RCT	*n* = 20 schools	Presence of an à la carte area in the school cafeteria operated by the school food service; a food service director and principal willing to take part in the study for two school years; an informed consent	School nutrition policy initiative	BMI-SD, height, overweight, obese	Weak
8	Fung et al.	2013	Canada	Cross-sectional	*n* = 5215(in 2003); *n* = 5508(in 2011)	All public schools were invited to participate	School food and nutrition policy (Children’s Lifestyle and School Performance Study-CLASS)	Dietary Status, Nutrient Intake, and Weight Status	Moderate
9	Gibson et al.	2008	USA	RCT	*n* = 4905 children (Intervention = 2505 and control = 2400)	Not mentioned	Physical Activity Across the Curriculum(PACC) - 90 mins moderate intensity physical activity delivered as part of academic instruction	Physical activity level	Weak
10	Hamilton et al.	2005	Australia	RCT	*n* = 4636 adolescents from 30 government high schools	Not mentioned	School-based smoking intervention(The Smoking Cessation for Youth Project-SCYP) - Curricular, parent, nurse counselling cessation support and policy components	The primary outcome variable was regular smoking(smoking on 4 or more days in the previous week) and the more traditional measure of ‘current smoking’ within the last 30 days was used for secondary analyses	Strong
11	Llargues et al.	2011	Spain	RCT	509 (Control: 237, Intervention: 272)	All the children born in 2000 who attended any of the schools in Granollers were eligible to participate	Teacher Training, Develop activities related to food habits and/or physical activity	Primary outcome: Difference in BMI progression Secondary outcomes: changes in eating habits and in physical activity	Strong
12	Knox et al.	2012	Australia	Quasi-experimental	182 pupils attending year (*n* = 115 Inter; 77 control)	Not mentioned	Physical Activity - Brisk Walking Lessons	Adiposity variables, BP, lipids, lipoproteins, glucose, insulin, high sensitivity C-reactive protein, high molecular weight adenopectin, aerobic fitness, physical activity behavior and diet	Weak
13	Jhonson et al.	2009	USA	Cross-sectional	*n* = 9151 students from 64 middle schools	All public schools that enroll seventh-grade students and participate in USDA school meal programs were eligible to participate	School district SSB policies	exposure of SSB and student consumption of SSB during the school days; school district policies about SSB and exposure to SSB in schools	Moderate
14	Lovato et al.	2006	USA	Cross-sectional	*n* = 522,318 students from 81 secondary schools	Not mentioned	School/ District tobacco control policies - scale for prohibition, strength, and characteristics of enforcement. Seven policy components: developing, overseeing and communicating the policy, purpose, and goals, prohibition, strength of enforcement, tobacco use prevention education and assistance to overcome tobacco addictions (Perception of policy)	Student smoking	Weak
15	Manios et al.	1999	Greece	RCT	*n* = 4171 students (Intervention); *n* = 1510 students (control)	All students in the first grade of selected schools	Multicomponent workbooks covering dietary issues, physical activity and fitness, dental health hygiene, smoking and accident prevention	Health Knowledge, Dietary, Physical Activity, Fitness, Anthropometric Measurements, Biochemical Indices	Moderate
16	Moore and Tapper	2008	UK	Randomized controlled trial	43 primary schools	The school was excluded if existing tuck shop, selling any food	Fruit Truck Shops	Purchase and Intake of Fruits	Moderate
17	Murnaghan et al.	2008	Canada	Cross-Sectional	*n* = 4709 grade 10 students	Not mentioned	Policy banning smoking in school property participated in provincially directed school-based smoking prevention program	Occasional and Current Smoking	Weak
18	O Brien et al.	2010	USA	Cross-Sectional	*n* = 80,428 students in 328 schools across the state of Maine. *n* = 123 intervention;205 non-intervention schools	Not mentioned	Comprehensive school health education, physical education and physical activity, school nutrition and food services, health promotion and wellness, school counselling physical and behavioral health services, school climate, physical environment, youth, parent, family and community involvement	Behavior change: physical activity, nutrition, and tobacco use	Weak
19	Paek et al.	2013	USA	Cross-Sectional	*n* = 983; 14 schools	All regular public schools containing grades 9, 10, 11, or 12 were included in the sampling frame	Tobacco-free school policy	Frequency of smoking	Weak
20	Patel et al.	2012	India	Cross-Sectional	*n* = 172 students from 2 schools	Male adolescents (aged 13-15 yrs.) not meeting the age criteria were excluded	Tobacco promotion and availability around schools	Current smoking and smokeless tobacco use	Weak
21	Schwartz et al.	2009	USA	Quasi- Experimental		Not mentioned	Removal of snacks of low nutritional value	Intake of beverages, salty snacks and sweet snacks	Weak
22	Spence et al.	2013	UK	Natural Experiment	*n* = 385 [2003–4]; *n* = 632 [2008–9]	Not mentioned	Nutrient-based standards	Mean daily intakes of macro & micro nutrients in school lunch packed lunch and total diet	Weak
23	Vandongen et al.	1995	USA	Cross-Sectional	1147 students from 30 schools	Not mentioned	Fitness, fitness + school nutrition, school-based nutrition, school + home nutrition, home-based nutrition	BP, Dietary Intake, 1.6 km run and Leger shuttle run, anthropometric measurements (subscapular skinfold) BMI, percentage fat	Weak
24	Raczynski et al.	2009	Australia	RCT	*n* = 2202	Not mentioned	Comprehensive legislation to combat obesity	BMI	Weak
25	Harris et al.	1997	USA	Quasi- Experimental	*n* = 170	Not mentioned	Modified school lunches, Enhanced nutrition education and increased opportunities for physical activities	Calorie intake, Physical fitness assessment, Knowledge, skills and attitudes related to nutrition and physical fitness	Weak
26	Holt et al.	2013	USA	Quasi- Experimental	Four elementary schools(grade k-5; 68 classroom teachers; 1284 students	Not mentioned	District mandated Physical Activity Policy (20 min)	The level of intensity of physical activity	Weak
27	Jaenke et al.	2012	Australia	Quasi- Experimental	*n* = 127 children (11-12 years)	Eligibility for participation in the study was for students to be enrolled in school Grades 5 or 6.	Nutrition education, gardening program	Food preference assessment, fruit and vegetable intake	Weak

### Physical and anthropometric measures

Seven studies assessed the effectiveness of policy interventions or its association with changes in anthropometric measures [14, 21–26]. Three studies assessed the effectiveness of school policy in controlling blood pressure [22, 26, 27]. The policy interventions targeted at anthropometric measurements (BMI, waist circumference, height and weight status) included school nutrition policy initiative [14, 21], comprehensive legislation at state level to combat obesity [25], brisk walking lessons [22], teacher trainings, developing activities related to food habits and/or physical activity [23], fitness guidance, fitness and school nutrition, school-based nutrition, school and home nutrition and home-based nutrition [26]; multicomponent workbooks covering dietary issues, physical activity and fitness [24] and integration of health promotion in the existing curriculum [27] (Table 3).

**Table 3:** Effectiveness of policies on physical, anthropometric measurements and biomarkers

Study	Design	Policy	Outcomes	Specific outcome	Impact (+) Favorable and significant change (=) No change	Strength of Association
*Physical and Anthropometric measurements*						
Foster et al., 2008 [14]	RCT	School self-assessment; nutrition education; nutrition policy; social marketing; and parent outreach.	BMI, Overweight, Obesity	BMI	=	Control (Baseline: 20.76 kg/m^2^; Follow up: 23.06 kg/m^2^) Intervention (Baseline 21.07 kg/m^2^; Follow up 23.06 kg/m^2^) *p*-value 0.71
				Overweight (BMI for age from the 85th to 94.9th percentile)	+	Adjusted Odds for Incidence:- 0.67 (0.47–0.96)
				Obesity (BMI for age _95th percentile)	=	Adjusted Odds for Incidence:- 1.00 (0.66–1.52)
Fung et al., 2013 [21]	Cross-sectional	School based nutrition policy	Overweight and Obesity (BMI measurements)	Overweight	=	Overweight (Prevalence ratio, adjusted change: 1.03 (0.94, 1.12)
				Obesity	+	Obesity (Prevalence ratio, adjusted change: 1.26 (1.08, 1.48))
Knox et al., 2012 [22]	Quasi-experimental	Brisk walking lessons	Waist circumference, Systolic Blood Pressure	Waist circumference	+	Prevalence of elevated waist circumference: Control:- 9.8% Intervention:- 6.9
				Systolic Blood pressure	+	Prevalence of elevated BP: Control:- 3.3% Intervention:- 0%
Llargues et al., 2011 [23]	RCT	Teacher trainings, Activities around food habits and physical activity	BMI progression after two years	BMI	+	Control (Baseline: 16.5 kg/m^2^ (16.7 kg/m^2^ to 17.5 kg/m^2^) Follow up: 18.3 kg/m^2^ (17.9 kg/m^2^ to 18.7 kg/m^2^)) Intervention (Baseline: 17.1 kg/m^2^ (16.7 kg/m^2^ to 17.5 kg/m^2^) Follow up: 17.9 kg/m^2^ (17.4 kg/m^2^to 18.4 kg/m^2^)
Manios et al., 1999 [24]	RCT	Multi-component workbooks – National policy for health education	BMI Progression	BMI	+	Control (Baseline: 24.4 kg/m^2^ Follow up: 32.8 kg/m^2^) Intervention (Baseline: 16.2 kg/m^2^ Follow up: 16.3 kg/m^2^) *p* = 0.001
Raczynski et al., 2009 [25]	RCT	Comprehensive legislation	BMI	BMI	=	Mean BMI: (Baseline: 28.8 kg/m^2^; 1 year follow up: 23.2 kg/m^2^; 2 year follow up: 25.7 kg/m^2^; 3 year follow up: 26.9 kg/m^2^) *p* value non-significant.
Vandongen et al., 1995 [26]	Cross Sectional	Guidance around fitness and nutrition (Fitness, Fitness + School Nutrition, School nutrition, School and home nutrition, home nutrition, All groups together)	BMI, Systolic blood pressure, Percentage body fat, Triceps skinfold, Subscapular skinfold	BMI	=	Means: BMI (Intervention Baseline: 18.0 kg/m^2^ (95% CI 17.8 kg/m^2^, 18.3 kg/m^2^) Follow up: 18.5 kg/m^2^ (18.0 kg/m^2^, 18.5 kg/m^2^) Control Baseline: 17.6 kg/m^2^ (16.9 kg/m^2^, 18.3 kg/m^2^) Follow up: 18.2 kg/m^2^ (17.4 kg/m^2^, 18.9 kg/m^2^)
				Systolic blood pressure	=	Systolic blood pressure (Intervention Baseline: 104.8 mm/Hg (104.0 mm/Hg, 105.9 mm/Hg) Follow up: 102.2 mm/Hg (101.4 mm/Hg, 104.9 mm/Hg) Control Baseline: 105.9 mm/Hg (104.1 mm/Hg, 107.7 mm/Hg) Follow up: 103.1 mm/Hg (101.3 mm/Hg, 106.5 mm/Hg)
				Percentage body fat	=	Percentage body fat (Intervention Baseline: 22.4% (21.9%, 23.0%) Follow up: 23.1% (22.5%, 23.7%) Control Baseline: 21.2% (19.6%, 22.8%) Follow up: 21.9%(20.3%, 23.6%)
				Triceps skinfold	+	Triceps skinfold (Intervention Baseline: 14.5 mm (14.0 mm, 14.9 mm) Follow up: 15.1 mm (14.5 mm, 15.6 mm) Control Baseline: 13.0 mm (11.9 mm, 14.1 mm) Follow up: 14.2 mm (12.6 mm, 15.7 mm))
				Subscapular skinfold	=	Subscapular skinfold (Intervention Baseline: 10.3 mm (9.8 mm, 10.8 mm) Follow up: 11.1 mm (10.5 mm, 11.6 mm) Control Baseline: 10.2 mm (8.7 mm, 10.8 mm) Follow up: 10.7 mm(9.1 mm, 12.2 mm))
Covelli, 2008 [27]	Quasi-experimental - Repeated Measures	Integration of health promotion into curriculum	Maintenance of blood pressure	Blood pressure	=	SBP: Intervention (Baseline 119.7 mm/Hg Follow up 116.2 mm/Hg) Control (Baseline 119.2 mm/Hg Follow up 119.1 mm/Hg; *p* = 0.56)
						DBP: Intervention (Baseline 66.2 mm/Hg Follow up 67.2 mm/Hg) Control (Baseline 66.8 mm/Hg Follow up 68.0 mm/Hg; *p* = 0.97)
*Biomarkers*						
Knox et al., 2012 [22]	Quasi-experimental	Brisk walking lessons	Blood levels of triglycerides, high density lipoprotein cholesterol, high density lipoprotein: total cholesterol, glucose	Triglycerides	+	Elevated Triglycerides (Control: - 2.5%; Intervention:-1.2%)
				High density lipoprotein cholesterol	+	Elevated high density lipoprotein cholesterol (Control: - 3. 7% vs. Intervention:-1.2%2.7%)
				High density lipoprotein: total cholesterol	+	High density lipoprotein cholesterol to total cholesterol ratio (mean + − SD: 2% ± 4% [confidence interval (CI)o.o5 = 1% to 2%>], t_80_ = −3.5, *p* = .001)
				Glucose	+	Glucose (−.1 ± .4 mmol/L *p* = .002)
Manios et al., 1999 [24]	RCT	Multi-component workbooks – National policy for health education	Serum level lipid changes	Total Serum Cholesterol	+	Intervention (Baseline 187.4 mg/dl Follow up 173.7 mg/dl) Control (Baseline 177.3 mg/dl Follow up 190.6 mg/dl; *p* = 0.001)

Mixed results were reported for the effects of school policies on BMI. Non-significant differences or associations with BMI for policy interventions were reported by three studies [14, 25, 26]; while two studies reported significantly lower progression of BMI among those exposed to policy interventions compared to those who did not [23, 24]. The studies that showed policies to be effective in reduction of BMI were assessed to have moderate and strong methodological quality. These effective policy interventions included teacher training, developing activities related to food habits and/or physical activity, multicomponent workbooks covering dietary issues, physical activity, and fitness. Decreased levels of elevated waist circumference as a result of brisk walking lessons was reported in one of the studies [22]; however, this study scored weak in quality assessment. Studies where case definitions included overweight and obesity also showed mixed results. While policy intervention of school nutrition policy initiative was effective in reduction of overweight in one study [14], an increase in the prevalence of both overweight and obesity was observed in another [21]. Though, the study showing effectiveness of school nutrition policy initiative had strong methodological quality.

Two studies that assessed the effectiveness of policies including brisk walking lessons and fitness guidance, fitness and school nutrition, school-based nutrition, school and home nutrition and home-based nutrition in BP control showed desirable effects [22, 26]. On the other hand, one assessing the effectiveness of integration of health promotion in the existing curriculum reported non-significant changes [27] (Table 3). However, the three studies were judged to be of weak methodological quality.

### Biomarkers

Two out of the 27 studies assessed changes in biomarker levels [22, 24]. One study [22] assessed whether extended brisk walking lessons as a school level intervention resulted in changes in serum levels of triglycerides, high-density lipoprotein cholesterol, high-density lipoprotein to total cholesterol ratio and glucose. They reported significantly lower levels of triglycerides, improvements in high-density lipoprotein cholesterol, high-density lipoprotein to total cholesterol ratio and reduction in glucose levels to be associated with the intervention. Similarly, desirable serum level lipid changes were reported by Manios et al. [26], in their study on the effectiveness of multicomponent workbooks covering dietary issues, physical activity and fitness, dental health hygiene, smoking and accident prevention as school level policies. Additionally, teaching aids including posters, audio-taped fairy tales for classroom use, workbooks, and teaching manuals were provided to class teachers and physical education (PE) instructors (Table 3). The studies were judged to have weak to moderate methodological quality.

### Unhealthy diet

The majority of selected studies (*n* = 15) assessed the effectiveness of policy interventions in changing unhealthy dietary behaviors. These policies ranged from removal of sugar-sweetened beverages (SSBs) and junk food [28–30]; change in canteen policies (increasing the availability of lower-fat foods in cafeteria’s à la carte areas and implementing school-wide, student-based promotions of these lower-fat foods) [31]; school self-assessment; nutrition education; nutrition policy (meet nutritional standards based on Dietary Guidelines for Americans); social marketing; and parent outreach [14]; fruit truck shops [32]; nutrition education and gardening program [33]; brisk walking lessons [22]; integration of health promotion in curriculum [27]; teacher trainings and development of activities related to food habits and/or physical activity [23]; fitness guidance, fitness and school nutrition, school-based nutrition, school and home nutrition and home-based nutrition [26]; modified school lunches, enhanced nutrition education and increased opportunities for physical activities [34]; comprehensive school health education, physical education and physical activity, school nutrition and food services, health promotion and wellness, school counselling, physical and behavioral health services, school climate, physical environment, youth, parent, family and community involvement [35]. Six out of seven studies assessing policy effectiveness in reduction of sugar intake reported desirable changes and reduction in sugar or SSBs consumption [21, 26, 28, 29, 35, 36]. These effective policies included elimination of SSB and other junk food in schools’ food policy, having a school food and nutrition policy in place, school district SSB policies, school nutrition and food services, nutrition-based standards and fitness guidance, fitness and school nutrition, school-based nutrition, school and home nutrition and home-based nutrition. Among these policy interventions, studies with moderate methodological quality evaluated school food and nutrition policy and school district SSB policies, while the remaining studies were judged to have weak methodological quality.

School policies were also observed to be effective in increasing fruit and vegetable intakes in four out of five studies [23, 27, 31–33]. Desirable effects of increased fruit and vegetable intakes were noted with the policy interventions of integration of health promotion in the curriculum, change in canteen policies, nutrition education and gardening program, teacher training and development of activities related to food habits and/or physical activity and fruit truck shops. Out of these effective interventions, teacher training and development of activities related to food habits and/or physical activity and fruit truck shops were observed to be reported from studies with moderate and strong methodological quality. Regarding fat reduction and salty snacks, school dietary policies were reported to reduce their prevalence [26, 30, 36] (Table 4). All the three studies were judged to have weak methodological quality.

**Table 4:** Effectiveness of policies on dietary behaviors

Study	Design	Policy	Outcomes	Specific outcome	Impact (+) Favorable and significant change (=) No change	Strength of Association
Blum et al., 2008 [28]	Quasi-experimental	Elimination from SSB (Diet) and other junk food in schools food policy	Sugar-sweetened beverages consumption	SSB	=	Consumption of SSB decreased in both intervention and control boys (F = 53.69, *P* < .05) and girls (F = 22.87, *P* < .05). Intervention girls decreased diet soda consumption as compared to control girls (F = 6.57, *P* < .05)
				Diet soda	=	
				Juice	=	
Johnson et al., 2009 [29]	Cross-sectional	School district SSB policies	Sugar-sweetened beverages consumption	SSBs	+	β = − 9.50, *p* < .0002
Schwartz et al., 2009 [30]	Quasi- Experimental	Removal of snacks of low nutritional value	Consumption of beverages, salty snack, and sweet snack	Beverages	+	β = −.23, *p* < .05
				Salty snack	+	β = −.30, *p* < .05
				Sweet snack	=	Not reported
French et al., 2004 [31]	RCT	School nutrition policy initiative: Food availability in à la carte areas; Peer promotions	Lower-fat food choices, Added fats score, Fruit and vegetable score	Lower-fat food choices	=	% Yes: Intervention (Baseline 0.29 First year 0.28 Second year 0.28) Control (Baseline 0.23 First year 0.26 Second year 0.24; *p* = 0.62)
				Added fats score	=	% Yes: Intervention (Baseline 2.5 First year 2.6 Second year 2.4) Control (Baseline 2.6 First year 2.7 Second year 2.5; *p* = 0.97)
				Fruit and vegetable score	=	% Yes: Intervention (Baseline 2.7 First year 2.9 Second year 2.9) Control (Baseline 2.8 First year 3.1 Second year 3.1; *p* = 0.95)
Foster et al., 2008 [14]	RCT	School self-assessment; nutrition education; nutrition policy; social marketing; and parent outreach.	Total energy consumed (kilo- joules), fat consumption (grams), and the number of fruit and vegetable servings	Energy	=	Adjusted difference: −104.27 (−234.28, 25.73) *p* = 0.12
				Fat consumption	=	Adjusted difference: −3.78 (−8.59, 1.02) *p* = 0.12
				Fruit and vegetable servings	=	Adjusted difference: −0.04 (−0.37, 0.3) *p* = 0.82
Fung et al., 2013 [21]	Cross-sectional	School food and nutrition policy (Children’s Lifestyle and School Performance Study-CLASS)	Fruit/vegetable, grain products, milk products, meat and alternatives, soda intake, SSBs,	Fruit/vegetable	=	β = −0.08 (−0.27, 0.19)
				Grain products	+	β = 0.26 (0.17, 0.34)
				Milk products	+	β = 0.24 (0.18, 0.31)
				Meat and alternatives	+	β = 0.06 (0.03, 0.09)
				Soda intake	+	β = −0.09 (−0.11, −0.06)
				SSBs	+	β = −0.20 (−0.27, −0.12)
Moore and Tapper, 2008 [32]	RCT	Fruit Truck Shops	Consumption of fruit and sweet and savoury snacks	Fruits	=	β = 0.06 (−0.1, −0.21)
				Sweets, Chocolates, Biscuits	=	β = −0.1 (−0.3, 0.01)
				Crisps	=	β = −0.05 (−0.2, 0.06)
Jaenke et al., 2012 [33]	Quasi- Experimental	Nutrition education, gardening program	Fruit and vegetable intake	Fruits	=	*P* = 0.93
				Vegetables	+	*P* = 0.67
Knox et al., 2012 [22]	RCT	Brisk Walking Lessons	Consumption of total fat, saturated fat, carbohydrates, proteins, fiber, and total calories	Consumption of total fat, saturated fat, carbohydrates, proteins, fiber, and total calories	=	Non-significant changes, effect, estimates not reported
Covelli, 2008 [27]	Quasi-experimental - Repeated Measures	Integration of health promotion in existing curriculum (Provision of cognitive behavioral components of health knowledge, health promotion concepts, nutrition, and exercise).	Fruits/vegetables per day	Fruits/vegetables per day	+	Fruits/vegetables per week: Intervention (Baseline 2.6 Follow up 4.9) Control (Baseline 2.7 Follow up 2.5; *p* = 0.0001)
Llargues et al., 2011 [23]	RCT	Teacher Training, Develop activities related to food habits and/or physical activity	Fruits, vegetables, SSBs, Sweets, Fizzy drinks	Fruits	=	No changes: Intervention 84.2% (*p* = 0.36) Control 80.0% (0.18)
				Vegetables	=	No changes: Intervention 73.1% (*p* = 0.58) Control 81.4% (0.84)
				Sweets	=	No changes: Intervention 93.7% (*p* = 1.0) Control 94.3% (0.7)
				Fizzy drinks	=	No changes: Intervention 87.9% (*p* = 1.0) Control 89.2% (0.12)
Vandongen et al., 1995 [26]	Cross-Sectional	Guidance around fitness and nutrition (Fitness, Fitness + School Nutrition, School Nutrition, School and home nutrition, home nutrition, All groups together	Fat, sugar and protein	Fat	Boys =Girls +	Fat % energy (Intervention Baseline: 33.1 (32.7, 33.7) Follow up: 33.7 (33.1, 34.3) Control Baseline: 33.2 (32.7, 33.7) Follow up: 33.2 (31.5, 34.9)
				Sugar	Boys + Girls =	Sugar % energy (Intervention Baseline: 22.8 (22.1, 23.5) Follow up: 21.9 (21.2, 22.7) Control Baseline: 21.7 (20.0, 23.3) Follow up: 23.3 (21.4, 25.3)
				Protein	Boys + Girls +	Protein (% energy) Intervention Baseline: 15.5 (15.3, 15.8) Follow up: 15.6 (15.2, 15.9) Control Baseline: 15.8 (15.1, 16.4) Follow up: 14.7 (13.9, 15.4)
Harris et al., 1997 [34]	Quasi- Experimental	Modified school lunches, Enhanced nutrition education and increased opportunities for physical activities	Knowledge and awareness regarding nutrition	Awareness levels	+	(t [33] = −6.64, *p* < .0001)
O’Brien et al., 2010 [35]	Cross-Sectional	Comprehensive school health education, physical education and physical activity, school nutrition and food services, health promotion and wellness, school counselling physical and behavioral health services, school climate, physical environment, youth, parent, family and community involvement	SSB consumption	Two or more sodas/week	+	OR: 0.83 (*p* = .023)
Spence et al., 2013 [36]	Natural Experiment	Nutrient-based standards	Non-milk extrinsic sugar	% energy NMES	=	Mean difference: −2.6 (−3.2, −1.9) (*p* < 0.001)

### Tobacco and alcohol use

Seven out of 27 studies assessed the effects of school tobacco control policies on the prevalence of tobacco use [20, 35, 37–41]. These school level tobacco control policies included comprehensive smoking bans, policy orientation towards abstinence and harm minimization principles, penalty on possession of tobacco products among students [37]; school-based smoking intervention: curriculum, parent, nurse counselling cessation support and policy components such as scale for prohibition, strength and characteristics of enforcement [38]. One study assessed seven policy components: developing, overseeing and communicating the policy, purpose, and goals, prohibition, the strength of enforcement, tobacco use prevention education and assistance to overcome tobacco addictions (perceptions regarding policy) [39]. Others assessed a policy banning smoking in school property [40], tobacco-free school policy [41], reduced tobacco promotion and availability around schools [20] and finally comprehensive school health education, school counselling, physical and behavioral health services, school climate, physical environment, youth, parent, family and community involvement [35] (Table 5).

**Table 5:** Effectiveness of policies on substance misuse (tobacco and alcohol use)

Study	Design	Policy	Outcomes	Specific outcome	Impact (+) Favorable and significant change (=) No change	Strength of Association
*Tobacco use*						
Evans-Whipp et al., 2010 [37]	Cross-sectional	Comprehensive smoking bans, policy orientation towards abstinence and harm minimization principles, possession of tobacco products among students	Current tobacco use, Daily smoking, Perception of smoking in school campus	Smoking ban – current smoking	=	OR (95% CI): 0.86 (0.59, 1.25)
				Harm minimization – current smoking	=	OR (95% CI): 1.09 (0.99, 1.21)
				Strict enforcement – current smoking	=	OR (95% CI): 0.78 (0.57, 1.05)
				Smoking ban – daily smoking	=	OR (95% CI): 0.95 (0.53, 1.69)
				Harm minimization – daily smoking	=	OR (95% CI): 1.01 (0.85, 1.20)
				Strict enforcement – daily smoking	=	OR (95% CI): 0.70 (0.44, 1.12)
				Smoking ban – perception	=	OR (95% CI): 1.39 (0.67, 2.89)
				Harm minimization – perception	=	OR (95% CI): 1.18 (0.97, 1.43)
				Strict enforcement – perception	+	OR (95% CI): 0.45 (0.25, 0.82)
Hamilton et al., 2005 [38]	RCT	School-based harm minimization smoking intervention	Regular smoking, smoking within previous month	Regular smoking	+	Intervention vs comparison OR (95% CI): Baseline 0.74 (0.45, 1.22) Post-intervention: 0.50 (0.33, 0.74)
				Smoking within previous month	+	Intervention vs comparison OR (95% CI): Baseline 0 0.95 (0.71, 1.28) Post-intervention: 0.69 (0.53, 0.91)
Lovato et al., 2007 [39]	Cross sectional	School/ District tobacco control policies – policy intention (written policy), policy implementation, perception of policy enforcement	Prevalence of smoking	Policy intention – smoking prevalence	+	β = −0.11 (R^2^ = 0.27) (*P* < 0.05)
				Policy implementation – smoking prevalence	+	β = −0.04 (R^2^ = 0.21) (*P* < 0.05)
				Policy perception – smoking prevalence	+	β = −0.55 (R^2^ = 0.62) (*P* < 0.05)
Murnaghan et al., 2008 [40]	Cross-sectional	Policy banning smoking in school property participated in provincially directed school-based smoking prevention program	Current smoking, Occasional smoking and Regular smoking	Occasional vs nonsmoker	=	OR (95% CI): 1.54 (0.79, 3.01)
				Regular vs occasional smoker	=	OR (95% CI): 0.92 (0.69, 1.23)
O’Brien et al., 2010 [35]	Cross-Sectional	Tobacco control school-based policy – Teacher shared information of consequences of smoking	Frequency of smoking in past month	Average cigarettes smoked/day	=	Not reported
Paek et al., 2013 [41]	Cross-sectional	Tobacco-free school policy	Frequency of smoking	Frequency of smoking	+	β = −0.56 (*P* < 0.05)
Patel et al., 2012 [20]	Cross-sectional	Avoiding tobacco promotion	Tobacco use	Tobacco use	+	*P* < 0.05
*Alcohol use*						
Evans-Whipp et al., 2013 [42]	Cross-sectional	Low policy enforcement, Abstinence alcohol message, Harm minimization alcohol message	Alcohol use: alcohol use on school grounds, current alcohol use. Binge drinking, student alcohol harm	Low policy enforcement - Use on school grounds	+	OR (95% CI): 1.48 (1.07, 2.05)
				Low policy enforcement - Current alcohol use	=	OR (95% CI): 1.12 (0.95, 1.32)
				Low policy enforcement - Binge drinking	=	OR (95% CI): 1.14 (0.94, 1.38)
				Low policy enforcement - Student alcohol harm	=	OR (95% CI): 1.02 (0.84, 1.25)
				Abstinence alcohol message - Use on school grounds	=	OR (95% CI): 0.85 (0.66, 1.10)
				Abstinence alcohol message - Current alcohol use	=	OR (95% CI): 0.90 (0.78, 1.04)
				Abstinence alcohol message - Binge drinking	=	OR (95% CI): 0.84 (0.59, 1.25)
				Abstinence alcohol message - Student alcohol harm	=	OR (95% CI): 0.86 (0.71, 1.04)
				Harm minimization alcohol message - Use on school grounds	=	OR (95% CI): 0.90 (0.73, 1.10)
				Harm minimization alcohol message - Current alcohol use	=	OR (95% CI): 0.92 (0.83, 1.02)
				Harm minimization alcohol message - Binge drinking	=	OR (95% CI): 0.82 (0.72, 1.92)
				Harm minimization alcohol message - Student alcohol harm	+	OR (95% CI): 0.83 (0.71, 0.96)

Current smoking was the preferred outcome of evaluation for tobacco use among four out of seven studies [20, 37, 38, 40] and smokeless tobacco use was measured as an outcome in only one of the seven studies [20]. Several other outcomes such as frequency of tobacco use, perception about school smoking and occasional smoking were also assessed in some of the included studies. Two studies reported non-significant differences between those exposed and not exposed to policy [35, 37] while five studies reported a significant reduction in tobacco use among those exposed [20, 38–41]. Among the effective interventions, only school based harm minimization smoking intervention was observed to be tested within a study with strong methodological quality. The remaining four studies scored weak in quality assessment. Only one study tested the association between school-level policies and alcohol use and reported that when the students believed the policy enforcement was not strict, the chances of students consuming alcohol on school grounds were higher [42] (Table 5). This study was judged to have moderate methodological quality.

### Physical inactivity

Among the 27 studies, ten assessed associations between school policies and changes in physical activity [22–24, 26, 27, 34, 35, 43–45]. The school policies included learning landscape program (renovation of school grounds) [43]; 90 min moderate intensity physical activity delivered as part of academic instruction [44]; lessons on brisk walking [22]; district mandated physical activity policy (20 min per day) [45]; integration of health promotion in existing curriculum (provision of cognitive behavioral components of health knowledge, health promotion concepts, nutrition and exercise) [27]; teacher trainings, developing activities related to food habits and/or physical activity [23]; fitness trainings [26]; increased opportunities for physical activities (installing physical fitness stations in each classroom; initiating a non-competitive incentive system based on students’ personal goals; training of PE teachers and lesson plans for PE teachers) [34]; comprehensive school health education, including physical education and physical activity, school nutrition and food services, health promotion and wellness, school counselling, physical and behavioral health services, school climate, physical environment, youth, parent, family and community involvement [35]; multicomponent workbooks covering dietary issues, physical activity and fitness, dental health hygiene, smoking and accident prevention [24]. All studies reported significant and positive changes in physical activity with the implementation of school policies except one [34] (Table 6). However, majority of these studies were of weak methodological quality and policies that were observed to be effective from moderate and strong methodological studies included teacher trainings, developing activities related to food habits and/or physical activity; and, multicomponent workbooks covering dietary issues, physical activity and fitness, dental health hygiene, smoking and accident prevention.

**Table 6:** Effectiveness of policies on physical inactivity

Study	Design	Policy	Outcomes	Specific outcome	Impact (+) Favorable and significant change (=) No change	Strength of Association
Anthamatten et al., 2011 [43]	Case-control	Physical Activity (learning landscape program) (Renovation of school grounds)	Utilization of school yards for Physical Activity	Overall utilization of school yards	+	Mean difference: 7.0 *p* = 0.003
Covelli, 2008 [27]	Quasi-experimental	Integration of health promotion in existing curriculum (Provision of cognitive behavioral components of health knowledge, health promotion concepts, nutrition, and exercise).	Exercise	Exercise per week	+	Intervention group: Mean Baseline (2.6 (SE:0.9) Follow up 4.5 (SE: 1.4))
						Control group: Mean Baseline (2.1 (SE(0.8) Follow up 2.2 (SE 0.1)) *p* = 0.001
Gibson et al., 2008 [44]	RCT	Physical Activity Across the Curriculum (PACC) - 90 mins moderate intensity physical activity delivered as part of academic instruction	Physical activity	Levels of physical activity	+	Intervention students 3.40 ± 0.02 vs control students 2.17 ± 0.03, *p* < 0.0001
Harris et al., 1997 [34]	Quasi-experimental	Modified school lunches, Enhanced nutrition education and increased opportunities for physical activities	Physical activity	Fitness levels	=	Pretest (18%) to post-test (29%) (*p* = 0.29)
Holt et al., 2013 [45]	Quasi-experimental	District mandated Physical Activity Policy (20 min)	Physical activity: Walk/run, Movement activity	Walk/run	+	Not reported
				Movement	=	Not reported
Llargues et al., 2011 [23]	RCT	Teacher Training, Develop activities related to food habits and/or physical activity	Physical activity	Walking to school	+	No changes: Control 83% Intervention: 73.4% *p* < 0.05
				Exercise	+	No changes: Control 74.2% Intervention: 76.4% *p* < 0.05
Manios et al., 1999 [24]	RCT	Multicomponent workbooks covering dietary issues, physical activity and fitness, dental health hygiene, smoking and accident prevention	Leisure time physical activity	Leisure-time MVPA	+	Intervention (Baseline 0.9) (Follow up 2.8) Control (Baseline 1.4) (Follow up 2.0)
O’Brien et al., 2010 [35]	Cross-sectional	Comprehensive school health education, physical education and physical activity, school nutrition and food services, health promotion and wellness, school counselling physical and behavioral health services, school climate, physical environment, youth, parent, family and community involvement	Physical activity	TV viewing >2 h/day	+	Odds ratio: 0.95 *p* = 0.28
Vandongen et al., 1995 [26]	Cross-sectional	Fitness, fitness + school nutrition, school based nutrition, school + home nutrition, home based nutrition	Fitness	Leger score	+	Means: Leger score (Intervention Baseline: 43.2 (41.9, 44.4) Follow up: 44.7 (43.2, 46.2) Control Baseline: 42.2 (38.6, 45.7) Follow up: 41.0 (37.2, 44.9)
				Run time (minutes)	+	Means: Run time (Intervention Baseline: 9.9 (9.8, 10.1) Follow up: 9.6 (9.4, 9.8) Control Baseline: 10.4 (9.9, 10.7) Follow up: 9.9 (9.4, 10.4)

### Policy effectiveness

In total, for 103 outcomes independently evaluated within these studies, 47 outcomes (46%) had significant desirable changes when exposed to the school policies. In terms of the frequency, these interventions included: school self-assessment (*n* = 1); nutrition education (*n* = 5); nutrition policy (*n* = 9); social marketing and parent outreach (*n* = 1); health education (*n* = 5); extended brisk walking lessons (*n* = 6); teacher trainings (*n* = 3); guidance around fitness or fitness training (*n* = 4); school district SSB policies (*n* = 1); integration of health promotion in curriculum (*n* = 2); creating opportunities for physical activity (*n* = 1); smoking bans, policy orientation towards abstinence and penalty on possession of tobacco (*n* = 1); school-based harm minimization intervention (*n* = 2); district or school-based tobacco control policies (*n* = 3); tobacco free school policy (*n* = 1); alcohol abstinence and harm minimization messages (*n* = 2); investment on school infrastructure to promote physical activity (*n* = 2); physical activity in curriculum (*n* = 1); district mandated physical activity policy (*n* = 1). However, the policy interventions observed to be effective after restricting the evidence from studies having strong and moderate methodological quality were fewer in numbers. These included teacher trainings, activities related to food habits and physical activity, and multicomponent workbooks for desirable outcomes on anthropometric measurements, and biomarkers. For the outcome of change in dietary behaviors, evidence was supportive for school district SSB policies, school food and nutrition policy. Evidence from strong and moderate methodological quality studies showed that school based harm minimization smoking intervention was effective for reduction of smoking, and low policy enforcement for alcohol use for the desirable outcome of reduced alcohol intake. Finally, for desirable changes in physical activity; the teacher trainings, developing activities related to food habits and/or physical activity; and, multicomponent workbooks were reported to be effective.

## Discussion

The current study systematically reviewed the evidence on the effectiveness of school policies in the reduction of risk factors for NCDs. Although the overall evidence indicates effectiveness of policies on behavioural outcomes and biomarkers, majority of these studies were judged to have weak to moderate methodological quality. Compared to these outcomes, school policies were evident to be less effective for the outcomes such as anthropometric measurements, overweight and obesity, and, alcohol use. None of the included studies reported long-term follow-up of participants. Therefore, it is not known whether beneficial changes in NCD risk factors resulting from school policy interventions are sustained in the longer term.

The NCD risk factors evaluated within the selected studies ranged from individual health behaviors to anthropometric measurements and biomarkers. The observed differences in the effectiveness of the policies according to these different types of outcomes may reflect the lag time between exposure to the intervention and effect on the outcome (NCD risk factor). For example, behavioral changes such as reduced sugar intake may be immediate following an intervention, but behaviors need to be sustained over a longer period to produce changes in anthropometric measures. These differences can also be due to the variations in the number of studies reporting these outcomes. While most selected studies tested the effectiveness of school policies on health behaviors (diet, smoking and physical activity) and anthropometric measurements, fewer studies tested associations between the school policies and biomarkers as well as between school policies and alcohol use.

High sugar consumption is associated with multiple NCDs including overweight/ obesity [46], diabetes [47] and dental caries [48]. The effective policy interventions targeted at the school level to reduce sugar consumption among school children were to have a school nutrition policy restricting SSBs. These findings are consistent with those reported in independent reviews on determinants of high sugar intake [49, 50]. Tobacco use is a well-established independent risk factor for NCDs and the most prevalent behavioral risk factor globally [51]. The most efficient school policy interventions included school-based harm minimization smoking intervention. These findings further substantiate the results on the positive influence of school environment on tobacco abstinence among children [52, 53]. Physical inactivity or sedentary behavior is also associated with worse health outcomes [54–56]. Teacher trainings, developing activities related to food habits and/or physical activity and, multicomponent workbooks were reported to be effective in increasing physical activity among school children from methodologically stronger studies. Another systematic review on school policy interventions and NCD risk factors reported the majority of the school policy interventions being effective [57].

This systematic review had several strengths and some limitations. A previous systematic review on the effectiveness of school policies was limited to the outcomes like physical inactivity, diet, and tobacco consumption [57] while the current review also included anthropometric measures, alcohol use, and biomarkers. Furthermore, the current systematic review included different study designs. Evidence suggests that RCTs are inappropriate for evaluating most health promotion interventions [58]. Thus, being inclusive of studies regardless of study designs allowed to present more comprehensive evidence on school policies. The review also had some limitations. First, the review was limited to English-language articles, which may have led to the exclusion of some relevant studies in other languages. Second, the review did not include grey literature including government reports due to lack of access. However, governments are likely to evaluate context specific school policy interventions which are less likely to be generalized to different contexts. Third, a meta-analysis of the evidence could not be conducted due to the heterogeneity among the policy components of the interventions included and the lack of uniformity in outcomes and effect measures. Finally, the evidence can only be summarized for the direction of association rather than its strength due to multiple heterogeneities within studies in terms of exposure, outcomes, and the effect estimates.

### Research and policy implications

The findings from the current review have important research and policy implications. The mixed evidence on the effectiveness of school policies according to different outcomes indicates that when planning intervention studies, the time lag between any policy exposure and the outcomes of interest should be considered carefully. Another important consideration is the duration of follow-up within studies; studies with longer-term follow-up of students are needed to assess whether positive changes in NCD risks are maintained beyond the intervention period and school years. Some of the school based policy interventions are expensive to design and resource intensive to carry out. The lack of good quality evidence on the effectiveness of school based policy interventions highlights the need for well-designed studies to inform the policymakers. Finally, all except one study were from high-income countries, which underscores the gap in research evidence from low- and middle-income countries. Global estimates indicate that 80% of the deaths due to NCDs occurs within low- and middle-income countries [59]. Thus more research on the effectiveness of school policies in the reduction of NCDs and their risk factors should also be conducted within these countries.

In most societies, NCDs are unequally distributed [60] and a shift in policy attention from individual to environmental and structural factors, such as the school environment, could be a more equitable approach [61]. The evidence from this systematic review will be used as a wider framework to aid in developing a contextually relevant and tailored comprehensive NCD intervention model for schools.

It will also guide the drafting of an evidence-based global school policies checklist for promoting a healthy lifestyle and preventing NCDs. The school checklist will inform policy-makers on aligning school curriculum, school activities and school health services, food availability and school infrastructure to be conducive for NCD prevention.

## Conclusions

Mixed findings were observed concerning effectiveness of school policies in reducing risk factors of NCD. More good quality evidence is required to conclude on the effectiveness of school level policies in reduction of NCD risk factors. Additionally, further research is required to assess whether healthy changes are sustained over long-term to reduce NCD risk in later life.

## Additional files

Additional file 1: Search strategy for Ovid Medline. (DOCX 34 kb)

Additional file 2: Table S1. Quality assessment of the selected studies. (DOCX 39 kb)

## Abbreviations

BMI: Body Mass Index
EPHPP: Effective Public Health Practice Project
LMIC: Low and Middle Income Country
NCD: Non-communicable Disease
PE: Physical Education
PRISMA: Preferred Reporting Items for Systematic Reviews and Meta-Analyses
RCT: Randomized Controlled Trial
SSB: Sugar Sweetened Beverage
