# Supplementary material for: Impact of school policies on non-communicable disease risk factors – a systematic review
Source: BMC Public Health. 2017 Apr 4;17:292. doi: 10.1186/s12889-017-4201-3 (PMC5379668; doi:10.1186/s12889-017-4201-3)
Supplement: Supplementary file 1 — Search strategy for Ovid Medline. (DOCX 34 kb) [file 12889_2017_4201_MOESM1_ESM.docx]

**Additional file 1**

**(**Search strategy for Ovid Medline)

Population

1. exp.child/
2. child*.mp
3. exp.adolescents/
4. adolescen*.mp
5. 1 or 2 or 3 or 4

Intervention

1. exp.policy/
2. policy or law* or bill* or legislat* or memorandum* or health promotion* or health behavi?r.mp
3. 6 or 7
4. impact* or intervention* or evaluat*.mp

Context

1. exp.school/

Outcome

1. exp.non communicable diseases/
2. exp Obesity/ or obes$.mp. or life style.mp. or exp Life Style/ or diet$.tw. or eat$.tw. or nutrition$.tw. or (physical adj1 activit$).tw. or exercise.tw. or play.tw. or tobacco.mp. or alcohol.mp.
3. 11 or 12

Overall

5 AND 9 AND 10 AND 13
