# Supplementary material for: Impact of school policies on non-communicable disease risk factors – a systematic review
Source: BMC Public Health. 2017 Apr 4;17:292. doi: 10.1186/s12889-017-4201-3 (PMC5379668; doi:10.1186/s12889-017-4201-3)
Supplement: Supplementary file 2 — Quality assessment of the selected studies. (DOCX 39 kb) [file 12889_2017_4201_MOESM2_ESM.docx]

Table S1 Quality assessment of the selected studies

| Study | Selection Bias (Overall) | Study Design (Overall) | Confounding (Overall) | Blinding (Overall) | Data Collection (Overall) | Withdrawal or dropouts (Overall) | Global Score  (Overall) |
| --- | --- | --- | --- | --- | --- | --- | --- |
| Anthamatten et al. | Weak | Moderate | Weak | Moderate | Strong | Weak | Weak |
| Blum et al. | Moderate | Moderate | Weak | Weak | Strong | Moderate | Weak |
| Covelli et al. | Weak | Strong | Weak | Moderate | Strong | Weak | Weak |
| Evans-Whipp et al. | Strong | Weak | Strong | Strong | Moderate | Not Applicable | Moderate |
| Evans-Whipp et al. | Strong | Weak | Moderate | Strong | Moderate | Not Applicable | Moderate |
| Foster et al. | Moderate | Strong | Strong | Strong | Strong | Moderate | Strong |
| French et al. | Weak | Strong | Weak | Strong | Moderate | Weak | Weak |
| Fung et al. | Strong | Weak | Strong | Strong | Strong | Not Applicable | Moderate |
| Gibson et al. | Moderate | Weak | Weak | Weak | Weak | Strong | Weak |
| Hamilton et al. | Strong | Strong | Strong | Moderate | Strong | Strong | Strong |
| Llargues et al. | Strong | Strong | Strong | Strong | Strong | Strong | Strong |
| Knox et al. | Moderate | Strong | Weak | Moderate | Moderate | Weak | Weak |
| Jhonson et al. | Moderate | Weak | Strong | Moderate | Strong | Moderate | Moderate |
| Lovato et al. | Strong | Weak | Weak | Moderate | Moderate | Strong | Weak |
| Manios et al. | Moderate | Strong | Strong | Weak | Strong | Strong | Moderate |
| Moore and Tapper | Strong | Strong | Strong | Weak | Strong | Strong | Moderate |
| Murnaghan et al. | Strong | Weak | Strong | Weak | Weak | Moderate | Weak |
| O Brien et al. | Moderate | Strong | Weak | Moderate | Weak | Weak | Weak |
| Paek et al. | Moderate | Weak | Strong | Moderate | Weak | Weak | Weak |
| Patel et al. | Weak | Weak | Weak | Weak | Weak | Not Applicable | Weak |
| Schwartz et al. | Weak | Weak | Moderate | Weak | Weak | Weak | Weak |
| Spence et al. | Moderate | Weak | Strong | Weak | Moderate | Weak | Weak |
| Vandongen et al. | Strong | Strong | Weak | Weak | Weak | Strong | Weak |
| Raczynski et al. | Moderate | Weak | Strong | Weak | Weak | Weak | Weak |
| Harris et al. | Moderate | Moderate | Weak | Weak | Moderate | Strong | Weak |
| Holt et al. | Moderate | Moderate | Weak | Weak | Weak | Weak | Weak |
| Jaenke et al. | Strong | Weak | Weak | Weak | Moderate | Strong | Weak |
